# The contribution of housing renovation to children’s blood lead levels: a cohort study

**DOI:** 10.1186/1476-069X-12-72

**Published:** 2013-08-27

**Authors:** Adam J Spanier, Stephen Wilson, Mona Ho, Richard Hornung, Bruce P Lanphear

**Affiliations:** 1 Department of Pediatrics, Penn State University Hershey Medical Center, HS83, 500 University Drive, P. O. Box 850, Hershey, PA 17033-0850, USA; 2 Department of Medicine, University of Cincinnati, 231 Albert Sabin Way, Cincinnati, OH 45267-0535, USA; 3 Cincinnati Children’s Hospital Medical Center, 3333 Burnet Avenue, Cincinnati, Ohio 45229-3026, USA; 4 Child and Family Research Institute, British Columbia Children’s Hospital, 950 West 28th Ave., Vancouver, BC, Canada V5Z 4H4; 5 Faculty of Health Sciences, Simon Fraser University, 8888 University Drive, Burnaby, B.C., Canada V5A 1S6

**Keywords:** Blood lead, Lead, Renovation, Repair

## Abstract

**Background:**

Routine renovation of older housing is a risk factor for childhood lead poisoning, but the contribution to children’s blood lead levels is poorly defined for children with lower exposure levels.

**Methods:**

We examined a prospective cohort of 276 children followed from 6 to 24 months of age. We conducted surveys of renovation activities and residential lead hazards and obtained blood lead level (B-Pb) every six months. We analyzed B-Pb in a repeated measures design using a mixed effects linear model.

**Results:**

Parent reported interior renovation ranged from 11 to 25% of housing units at the four, 6-month periods. In multivariable analysis, children whose housing underwent interior renovation had a 12% higher mean B-Pb by two years of age compared with children whose housing units were not renovated (p < 0.01). The time between renovation and the child blood lead sample was associated with higher B-Pb (p-value for trend <0.01); compared to children in non-renovated housing, children whose housing units underwent renovation in the prior month had a 17% higher mean B-Pb at two years of age, whereas children whose housing renovation occurred in the prior 2–6 months had an 8% higher mean B-Pb. We also found an association between higher paint lead loading, measured using an X-ray fluorescence (XRF) based paint lead index, and child B-Pb (p = 0.02); for every 10 mg/cm^2^ increase in paint lead loading index there was a 7.5% higher mean childhood B-Pb.

**Conclusions:**

In an analysis of data collected before the recent changes to Environmental Protection Agency’s Lead, Renovation, Repair and Painting Rule, routine interior housing renovation was associated with a modest increase in children’s B-Pb. These results are important for the provision of clinical advice, for housing and public health professionals, and for policymakers.

## Background

Childhood lead exposure, even at low blood lead levels, is an established risk factor for intellectual deficits, conduct disorder, ADHD, renal injury, dental caries, and other health problems [1-10]. The blood lead level (B-Pb) that is classified by the Advisory Committee on Childhood Lead Poisoning Prevention of the Centers for Disease Control and Prevention (CDC) as elevated – the “level of concern” – has gradually been lowered over the past four decades, and the “reference value” is now 5 μg/dL [11]. Numerous commentators have argued for a shift to prevention of exposure to lead hazards (primary prevention), and the CDC recently recommended abolishing the term “blood lead level of concern,” due to extensive evidence indicating that there is no safe level of lead exposure [12-16].

Residential lead hazards, such as deteriorated lead-contaminated house paint, house dust, soil, and water, are the primary sources of childhood lead intake, especially for children who live in older housing. In 2002, it was estimated that over 25% of the nation’s housing units had one or more lead hazards; 68% of homes built before 1940 had lead hazards [17].

Routine home renovations can increase a child’s risk for lead poisoning. In a retrospective case control study, investigators noted that renovation and repair work was associated with a 10% increase in the risk of children having a B-Pb > 10 μg/dL [18]. Another study reported an association of home refinishing (i.e. sanding, scraping, or painting) with a 1.4 μg/dL increase in B-Pb; the increase was higher for children who lived in homes with higher lead paint levels [19]. While there is evidence that renovation is a risk factor for lead poisoning, few studies have prospectively examined the contribution of renovation activities to children’s B-Pb [19]. Moreover, few of the studies evaluating the impact of renovation on children’s blood lead levels studied children who had lower levels of B-Pb typically found in children today. The purpose of this study was to quantify the contribution of renovation to child B-Pb during the first two years of life, accounting for other major sources of exposure.

## Methods

Children and their families in this analysis were participants in a prospective cohort (the Rochester Lead Study) that included a randomized, controlled trial of dust control described previously [20,21]. Briefly, we enrolled 276 children who were age 5 to 7 months at baseline (born between July 1994 and January 1995), were living in Rochester, New York, and had no plan to relocate within three months of enrollment. A study team visited the home if the family was eligible, obtained consent, collected a child blood sample, conducted an extensive interview, and collected environmental samples. Cincinnati Children’s Hospital Medical Center Institutional Review Board approved this analysis.

We measured lead in children’s blood at baseline and 6 month intervals until 24 months of age (i.e., 6 months, 12 months, 18 months, and 24 months). Trained phlebotomists collected venous samples using techniques to minimize external contamination. We determined B-Pb using Electrothermal Atomization Atomic Absorption Spectrometry (New York State Department of Health, Wadsworth Laboratories, Albany, NY). The blood lead concentrations were the mean of six separate analyses of each sample (three measures on two consecutive days). The limit of detection for lead in blood was 1.0 μg/dL (1.9% of data were missing and 0.5% were below the limit of detection).

A trained research assistant conducted extensive surveys to assess residential lead hazards, renovation activities, and demographic factors at each visit (every six months). Interior renovation was defined as a positive response to any of the following three questions: “Has any sanding or scraping been done inside your home since our last visit”; “Have any windows been replaced in your home since our last visit”; and, “Have any walls, or ceilings been replaced inside your home since our last visit?” We also examined the impact of timing on renovation on children’s blood lead concentrations. At each visit, participants were classified as not having had interior renovation, having renovation in the past month or having renovation in the previous two to six months. Similarly, we defined exterior renovation as a positive response to any of the following three questions: “Has any exterior work such as scrapping or sanding on the walls or windows on the outside of your home occurred since our last visit;” “Has the porch been sanded, scraped, or painted since our last visit;” and “Have any of your next door or backyard neighbors sanded or painted outside their home since our last visit.”

A trained environmental technician collected dust samples at each visit using a standardized and validated protocol [22]. Briefly, the technician collected three or four composite wipe samples of house dust from floors, window sills, and window troughs or wells. Each composite wipe sample consisted of a maximum of three wipe samples collected from the same surface type (e.g. floors). We quantified the amount of lead in the composited wipe samples using flame atomic absorption, followed by graphite furnace if levels were below 5 μg per sample. The limit of detection for lead using the graphite furnaceon wipe samples was 0.5 μg (<0.1% of samples were below the limit of detection).

We measured lead content of paint on wall surfaces at up to 10 locations (entryway molding, entryway walls, kitchen window molding, kitchen walls, living room window molding, living room walls, bedroom window molding, bedroom walls, interior porch molding, interior porch floor) with a portable X-ray fluorescence lead-based paint analyzer (XRF, Microlead I, Warrington, Austin, TX). We calculated the mean of 3 measurements for each location, and the mean was adjusted (multiplied) by paint condition (range 1 to 3, with 3 being bad). We then calculated the mean of the 10 locations as an XRF based index of paint lead loading and paint condition.

The technician also collected soil and water samples at baseline for measurement of lead concentration. Three soil samples were collected from the foundation of the house where bare soil was present to create a composite measure. Research technicians collected water from the kitchen tap in the morning. They collected 250 mL of water after the one minute of water flow through an open tap. We measured soil lead concentration using flame atomic absorption spectroscopy, and the limit of detection was 25 μg/g. Water lead concentration was measured using atomic absorption with a limit of detection was 5 μg/L.

### Statistical analysis

We first calculated descriptive statistics for all demographic, exposure, and outcome data. We used a log transformation of B-Pb data because B-Pb was approximately log-normal and to minimize the influence of outliers [21]. SAS Version 9.2 (SAS Institute, Inc., Cary, NC) was used for all data analyses.

We analyzed the association of renovation activities with the continuous outcome variable B-Pb using a mixed effects linear model with subjects (children) as a random effect and subject characteristics as fixed effects. We first conducted bivariate analyses to evaluate the association of interior renovation and potential covariates with B-Pb. After bivariate analysis, we conducted a multivariable analysis. We used information from previous analyses of the contribution of residential lead hazards to children’s blood lead levels as a guide in the choice of variables for the multivariable analysis [21]. We also considered additional variables in the multivariable analysis if their p value was ≤ 0.2 in bivariate analysis. We retained those covariates if they were significant (p < 0.05) or if their addition caused a greater than 10% change in the estimate for B-Pb. In all analyses (including the bivariate analyses), we forced the inclusion of a variable for time point (6, 12, 18, 24 months) and a variable for the dust control intervention arm to account for any potential design effects of the embedded trial. We also explored whether race, age, iron intake, or calcium intake modified the relationship of renovation with B-Pb.

In a separate analysis, we evaluated effects of paint lead loading and condition (XRF index) in conjunction with renovation activity as well as potential interactions of renovation and paint lead concentration. In these analyses, we replaced floor dust and window well dust with the index of paint lead loading and condition.

In another separate analysis to explore potential associations of renovation with floor dust lead, we evaluated the association of renovation activity with the continuous variable log transformed floor lead loading using a mixed effects linear model. In this analysis, we included the XRF lead index variable to account for the paint lead in the setting of renovation.

## Results

A majority of the children who were enrolled in the Rochester Lead Study were Black, from lower income households, lived in rental property, and were from single parent households (Table 1). The frequency of any interior renovation activities ranged from a low of 11% to a high of 25% for each of the four 6-month survey periods (Table 2). The most common renovation activity was interior sanding or scraping, followed by wall or ceiling replacement. Renovation activities were more common in owner-occupied housing than in rental housing.

**Table 1:** **Baseline characteristics of children** &**their families (n = 276)**

**Characteristic**	**Number (%)**
Race	
Black	166 (60)
White	54 (20)
Latino	29 (10.5)
Asian	4 (1.4)
Other or unknown	23 (8.4)
Household income	
< $15,500	194 (71)
≥ $15,500	78 (29)
Rental housing	
Rent	235 (86)
Own	38 (14)
Marital status	
Single	161 (58)
Married	71 (26)
Single, living together	26 (9)
Divorced, separated or widowed	18 (7)

**Table 2:** Blood lead concentration, environmental lead level (95% CI) and renovation activities at six month intervals during early childhood

	**Participant age**			
	**6 months**	**12 months**	**18 months**	**24 months**
Blood lead (μg/dL) *	2.9 (2.7, 3.1)	5.7 (5.3, 6.2)	6.1 (5.6, 6.6)	7.5 (7.0, 8.2)
Blood lead ≥10μg/dL	1.4%	16.9%	22.7%	33.3%
Blood lead ≥5μg/dL	15.6%	63.8%	65.6%	74.8%
Floor dust lead (μg/ft^2^)	12.6 (10.9-14.3)	7.6 (6.5-8.7)	7.8 (6.5-9.0)	8.4 (7.0-9.7)
Sill dust lead (μg/ft^2^)	2,422 (873–3,971)	936 (427–1,444)	633 (405–861)	733 (294–1,172)
Trough dust lead (μg/ft^2^)	70,662 (58,581-82,743)	14,477 (11,002-17,952)	13,356 (10,425-16,287)	14,473 (10,752-18,194)
Soil lead (μg/g)	1,712 (1,470-1,954)	1,536 (1,295-1,777)	1,583 (1,340-1,826)	1,635 (1,379-1,890)
Paint lead (mg/cm^2^)	5.0 (4.2-5.7)	4.9 (4.1-5.8)	4.9 (4.1-5.8)	5.3 (4.3-6.2)
Any interior renovation, N (%)	60 (22.5)	62 (23.6)	62 (24.5)	27 (10.8)
Interior, sanding or scraping	43 (15.9)	39 (14.8)	42 (16.6)	15 (6.0)
Window replacement	18 (6.7)	17 (6.5)	13 (5.1)	14 (5.6)
Wall or ceiling replacement	18 (6.7)	24 (9.1)	26 (10.2)	12 (4.8)
Any exterior renovation	61 (26.6)	71 (28.0)	25 (10.4)	25 (10.6)

* All lead values presented are geometric means with 95% Confidence Intervals.

In bivariate analysis, we found that interior renovation was associated with higher B-Pb (p = 0.02). We also found that an increase in the time that elapsed since interior renovation was completed (having renovation in the past month, having renovation in the previous two to six months, and no renovation) was associated with decreasing B-Pb (p = 0.04). In contrast, exterior renovation was not associated with B-Pb (p = 0.57). We also evaluated each of the renovation activities separately (any sanding or scrapping, any window replacement, and any walls or ceilings replacement), but the frequency of the individual renovation activities other than sanding or scraping of paint was less than 10% at each visit. Scraping of paint had a marginal association with higher B-Pb (p = 0.1), but replacement of windows (p = 0.18) and replacement of walls or ceilings (p = 0.54) were not significantly associated with B-Pb.

**Figure 1:**
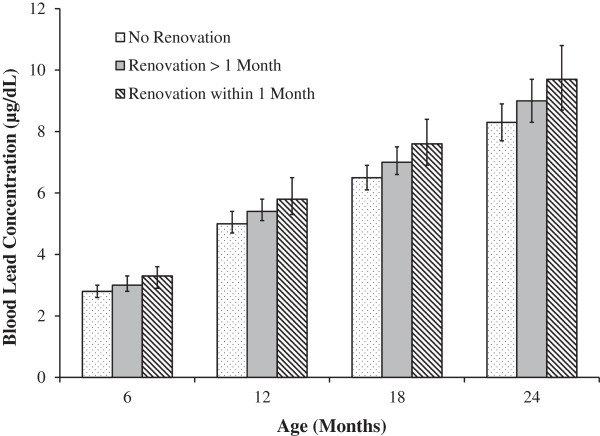
Adjusted blood lead concentration by interior renovation activities at each time point.

In multivariable analysis, children who lived in housing that underwent interior renovation had a 12% higher mean B-Pb by two years of age than children whose housing did not undergo interior renovation (Table 3, p < 0.01). For a child with a blood lead concentration of 5 μg/dL, this percent change would equate to a 0.6 μg/dL increase in mean B-Pb. The timing of renovation (no renovation, renovation in the previous 2–6 months, and renovation in the last month) was also associated with higher mean B-Pb (Figure 1, p-value for timing trend <0.01). Compared with children whose housing was not renovated, children whose housing underwent renovation in the previous month had a 17% higher mean B-Pb at two years of age, whereas children whose housing renovation occurred in the previous 2–6 months had an 8% higher mean B-Pb. In multivariable analysis exterior renovation was not associated with B-Pb (without interior renovation in the model p = 0.67, with interior renovation in the model p = 0.97). Additionally, removing the floor dust variable did not change the estimate (beta) for the renovation variable. There was no significant interaction of interior renovation and race (p = 0.75) in the analyses. Similarly, there was no statistically significant interaction of interior renovation and child’s age (p = 0.78), interior renovation and dietary iron intake (p = 0.54), or interior renovation and calcium intake (p = 0.97).

**Table 3:** Adjusted change in blood lead concentration (μg/dL) associated with sources of environmental lead, behaviors, dietary iron intake, and demographic characteristics from 6 to 24 months of age*

**Variables**		**Change in blood lead concentration**				
	**Estimate (standard error)**	**5**^**th **^**percentile**	**95**^**th **^**percentile**	**Percent change**	**Absolute change (μg/dL)†**	**P value**
Floor lead (μg/ft^2^)	0.014 (0.003)	0.8	30.7	39.4	1.97	<.0001
Soil lead (ppm)	0.002 (0.001)	0.18	46.6	11.3	0.57	0.02
Water lead (> 5 ppb)	0.186 (0.052)			20.4	1.02	<0.001
Time spent outdoors	0.090 (0.034)			9.4	0.47	0.009
Soil ingestion	0.143 (0.037)			15.3	0.77	<0.001
Interior renovation	0.115 (0.037)			12.2	0.61	0.002
*Age-modified variables*						
Iron intake (mg/day)	0.011 (0.004)					0.003
6 mo		5.6	39.4	−30.7	−1.54	
12 m		4.9	31.0	−0.8	−0.40	
18 mo		4.4	18.1	2.3	0.12	
24 mo		5.0	20.0	7.7	0.38	
Rental housing	−0.346 (0.089)					<0.001
6 mo				−8.6	−0.43	
12 mo				16.2	0.81	
18 mo				33.7	1.68	
24 mo				47.7	2.38	
Ingest paint chips	−0.246 (0.094)					0.009
6 mo				37.8	−1.89	
12 mo				16.2	0.81	
18 mo				5.1	1.68	
24 mo				−2.1	2.38	
Trough lead (μg/ft^2^)	0.019 (0.009)					0.044
6 mo		0.034	23.20	−9.4	−0.47	
12 mo		0.005	4.69	4.1	0.21	
18 mo		0.005	4.41	7.4	0.37	
24 mo		0.002	6.52	15.1	0.75	
Black race	0.179 (0.067)					0.007
6 mo				27.3	1.36	
12 mo				44.1	2.21	
18 mo				55.0	2.75	
24 mo				63.2	3.16	

* The percent change that is presented represents the change a child would incur if they increased their exposure from the 5^th^ percentile of a given exposure to the 95^th^ percentile; for age modified variables the percent change is presented for the specific 6 month measure. The variables with no presented value in the 5^th^ and 95^th^ columns were dichotomous, thus there is no 5^th^ or 95^th^ percentile.

† The absolute change that is presented represents the change a child with a blood lead concentration of 5 μg/dL would incur for a given exposure, behavior, or characteristic.

We also considered whether the paint lead loading and condition index measured using X-ray fluorescence (XRF) was associated with children’s B-Pb in a secondary analysis. As noted in the methods, we used a composite XRF variable which incorporated paint condition from up to 10 sites. We had had 274 paint (XRF) measurements for visit 1, ranging from 5 to 10 locations (mean number of locations measured = 7.5). We had 89 paint (XRF) measurements for visit 2, ranging from 4 to 10 locations (mean number of locations measured = 7.0). We had 79 paint (XRF) measurements, ranging from 3 to 10 locations for visit 3 (mean number of locations measured = 6.1). We had 73 paint (XRF) measurements for visit 4, ranging from 2 to 8 locations (mean number of locations measured = 5.0). In the multivariable analysis, we found that an increasing paint lead loading and condition index (condition adjusted XRF variable range 0–59 mg/cm^2^) was associated with a higher child B-Pb (p = 0.02). For every 10 mg/cm^2^ increase in the paint lead loading and condition index there was a 7.5% higher mean B-Pb. There was no significant interaction of paint lead loading and renovation activity (p = 0.32).

In bivariate analysis, there was no association of interior renovation with floor lead loading (p = 0.25), but the geometric mean floor lead level was slightly higher in the unrenovated housing units than the housing units which had been renovated across each study visit (data not shown). We also found there was no association with floor lead loading when we considered the time that elapsed since interior renovation was completed (p = 0.18), but the geometric mean floor lead level was highest for the floors in which there was reported renovation activity within the previous month followed by no renovation and renovation within the previous 2–6 months (Table 4). The addition of the XRF index did not affect either of these associations, and the interactions of the XRF index variable and renovation variables was not significant.

**Table 4:** Floor dust lead levels by renovation status at each visit

**Renovation**	**visit 1**		**visit 2**		**visit 3**		**visit 4**	
	**Mean (95% CI)**	**N (%) > 5 μg/ft**^**2**^	**Mean (95% CI)**	**N (%) > 5 μg/ft**^**2**^	**Mean (95% CI)**	**N (%) > 5 μg/ft**^**2**^	**Mean (95% CI)**	**N (%) > 5 μg/ft**^**2**^
**None**	7.3 (6.3,8.3)	117 (60.6)	4.7 (4.2,5.4)	83 (43.5)	4.6 (4.0,5.2)	81 (43.6)	4.5 (3.9,5.2)	109 (50.5)
**Yes**	6.6 (5.5,7.9)	28 (49.1)	4.3 (3.7,5.1)	19 (31.2)	4.2 (3.5,4.9)	21 (33.9)	4.1 (3.3,5.0)	10 (38.5)
**Renovation timing**								
**None**	7.2 (6.3,8.3)	117 (60.6)	4.7 (4.2,5.4)	83 (43.5)	4.6 (4.0,5.2)	81 (43.6)	4.5 (3.9,5.2)	109 (50.5)
**>1 month prior**	6.2 (5.0,7.6)	14 (41.2)	4.0 (3.4,4.8)	15 (37.5)	3.9 (3.2,4.7)	11 (26.2)	3.8 (3.1,4.8)	6 (31.6)
**<1 month prior**	7.4 (5.9,9.3)	14 (60.9)	4.9 (3.9,6.1)	4 (19.1)	4.7 (3.7,5.9)	10 (60.0)	4.6 (3.6,6.0)	4 (57.1)

## Discussion

We found that interior housing renovation was associated with a 12% increase in children’s B-Pb. The timing of renovation was also important; renovation activity that occurred closer in time to the measurement of B-Pb was associated with 17% higher mean B-Pb whereas renovations that occurred more remotely were associated with an 8% higher mean B-Pb. In contrast, exterior renovation activity was not a significant risk factor for changes in B-Pb in this analysis. The increase in blood lead concentrations, which has previously been shown to be due to contamination of house dust and inadequate clean-up after renovation, is modest for an individual child, but it can have substantial consequences at the population level.

Previous investigators have reported varying effects of renovation and lead abatement on children’s B-Pb. Amitai et al., reported that, “residential de-leading,” or abatement, caused an increase in B-Pb among children [23]. Farfel et al., reported that traditional abatement led to a 10 to 100 fold increase in house dust lead loadings; nearly half of children living in abated dwellings had an increase in their B-Pb [24]. Similarly, Aschengrau et al., reported that residential lead abatement was associated with an increase in B-Pb within the year following remediation for children with mildly elevated B-Pb [25]. Results from these and other studies ultimately led the United States Department of Housing and Urban Development’s (HUD) to promulgate a post-abatement clearance standard in 1999 to protect children from lead abatement. Subsequently, the Residential Lead-Based Paint Hazard Reduction Act of 1992 (Title X of the 1992 Housing and Community Development Act) mandated that the US Environmental Protection Agency (EPA) promulgate a health-based dust lead standard to protect children from lead hazards, including from renovation of older housing.

Interestingly, although we found that renovation was associated with children’s B-Pb levels, it was not associated with floor lead loading. There are several possible reasons for this result. First, floor lead levels were composite samples and may not represent the rooms in which the renovation occurred. Second, we do not have data on the extent of renovation or whether “lead safe” practices that took place during the renovation. The fact that the lowest floor lead loading levels occurred among homes in which renovation occurred two to six months prior to the lead collection appears to be paradoxical. But, this may reflect the observation noted by other investigators that renovation results in a transient increase in dust lead hazards and blood lead concentrations [24,25]. Our results suggest that children’s blood lead levels are better longer-term indicators of exposures generated by renovation than lead-contaminated house dust.

What clearance standards should be used after housing renovation to protect children? Several studies have found that existing residential dust lead standards – including the 40 μg/ft^2^ floor standard used by US EPA and HUD – are inadequate to protect children from lead hazards, even when a blood lead concentration > 10 μg/dL was considered unacceptable [12,26,27]. In the most striking example, Clark et al., found that 9.3% of children in homes with lead intervention had and the youngest children were at highest risk: 6-month old children were 11-times more likely than 42-month old children to have an increase in blood lead level > 5 μg/dL following lead hazard controls, indicating that it is critical to achieve lower clearance levels or improve hazard control after disrupting lead based paints [28]. More recently, Dixon et al. analyzed data from the National Health and Nutrition Examination Survey 1999–2004 and demonstrated that clearance level for floor dust should be significantly below the existing standards [29]. These studies provide clear and compelling evidence about how renovation and lead abatement can result in an increase in children’s blood lead concentrations, and supports regulations to require contractors to use evidence based hazard control and clearance standards to protect at risk populations.

These and other studies have contributed to the call for primary prevention efforts – particularly the elimination of lead hazards after abatement and renovation or before occupancy [12-14,30,31]. A recent cost-benefit analysis found that for every $1 invested in reducing lead exposure in housing, society would benefit by $17 to 220, a cost-benefit ratio that is better than vaccines for developed countries [32].

We found that routine home renovation was associated with a modest increase in B-Pb. These findings support efforts to regulate routine renovation practices of older homes occupied by young children to prevent lead toxicity. In 2011, the US EPA promulgated a regulation to protect children from renovation of older housing [33]. The EPA Lead, Renovation, Repair and Painting Rule requires that contractors who perform renovation, repair, or painting projects that could disturb lead-based paint in pre-1978 homes, child care facilities and schools be certified by EPA and follow lead-safe work practices. Unfortunately, the US EPA failed to require post-renovation clearance testing. Instead, they relied on a non-validated, cloth wipe (i.e. a “white glove test”) which is compared to standard pictures approximating the cleanliness of the surface. In contrast with the wipe sampling method, which has undergone extensive validation, the screening characteristics of the white glove test have not been thoroughly evaluated. The rule should require clearance testing based on empirical evidence after renovations and repair of painted surfaces.

There are some limitations to our analysis. First, the renovation activity was based on self-report; there was no information on the extent or duration of renovation. Information on the extent and duration of renovation would have permitted a greater differentiation in actual levels of exposure and risk. A second limitation is that we may not have directly measured the lead hazards of the room(s) in which the renovation occurred. We did have composite measures (from three rooms in the house) of paint lead loading and condition (XRF index) and dust lead loading, but they did not necessarily include the room or rooms that were renovated limiting our ability to directly assess the association of renovation with dust lead levels. A third limitation is that our results may not be generalizable to older housing units that undergo abatement or extensive renovations. Fourth, we do not have any information regarding whether the renovation was conducted in a lead safe manner, whether clearance testing was done, or the type of cleaning immediately after renovation. Finally, our results provide an estimate of the average increase in blood lead concentrations; the increase may be higher or lower for individual children.

## Conclusions

We found that interior renovation was associated with a modest but statistically significant increase in children’s blood lead concentrations. The relationship was stronger for children who had blood samples collected in the month immediately after the renovation was done. Clinicians should advise parents that routine renovation and repair of older housing generates lead hazards and should be conducted using lead-safe work practices and clearance testing to prevent children’s exposure. It is critical that EPA complete its reassessment of the dust lead standard and ensure that validated wipe sampling tests are used and dust clearance levels are achieved to protect young children from lead hazards [12,26,27,29]. Unfortunately, until empirically-based dust clearance standards are required, many children will inevitably and unnecessarily be exposed to lead hazards following renovation or abatement of older housing.

## Abbreviations

B-Pb: Blood lead level; CDC: Centers for disease control and prevention; CI: Confidence interval; EPA: Environmental protection agency; HUD: Housing and urban development; XRF: X-ray fluorescence.

## Competing interests

The authors have no competing financial or non-financial interests to disclose.

## Authors’ contributions

AS and BL developed the research question, jointly provided oversight over the entire analysis, and drafted and revised the paper. MH is guarantor of data analysis and contributed to the revisions of the paper. RH helped develop the data analytic plan, supervised the analysis, and contributed to the revisions of the paper. SW participated in the data analysis and evaluation and the drafting and revision of the paper. All authors participated in the discussion and interpretation of the results, critically revised the manuscript for intellectual content and approved the final version.
